# Utilizing a Novel 2D Image Processing System for Relating Body Composition Metrics to Performance in Collegiate Female Rowers

**DOI:** 10.3390/ijerph18052413

**Published:** 2021-03-02

**Authors:** Michael R. Esco, Clifton J. Holmes, Katherine Sullivan, Bjoern Hornikel, Michael V. Fedewa

**Affiliations:** Exercise Physiology Laboratory, Department of Kinesiology, University of Alabama, Tuscaloosa, AL 35487, USA; Clifton@email.wustl.edu (C.J.H.); ksullivan8@crimson.ua.edu (K.S.); bhornikel@crimson.ua.edu (B.H.); mvfedewa@ua.edu (M.V.F.)

**Keywords:** body fat, fat mass, fat-free mass, female athletes, rowing, technology

## Abstract

The purpose of this study was to determine if rowing performance was associated with fat mass (FM) or fat-free mass (FFM) measured using a novel 2D digital image analysis system. Nineteen female rowers (ages = 20.3 ± 1.0 years, weight = 73.8 ± 8.3 kg, height = 172.7 ± 4.7 cm) participated in this study. FM and FFM were estimated with a smartphone application that uses an automated 2D image analysis program. Rowing performance was measured using a 2 km (2k) timed trial on an indoor ergometer. The average speed of the timed trial was recorded in raw units (m·s^−1^) and adjusted for body weight (m·s^−1^·kg^−1^). FFM was significantly correlated to unadjusted 2k speed (r = 0.67, *p* < 0.05), but not for FM (r = 0.44, *p* > 0.05). When 2k speed was adjusted to account for body weight, significant correlations were found with FM (r = −0.56, *p* < 0.05), but not FFM (r = −0.34, *p* > 0.05). These data indicate that both FM and FFM are related to rowing performance in female athletes, but the significance of the relationships is dependent on overall body mass. In addition, the novel 2D imaging system appears to be a suitable field technique when relating body composition to rowing performance.

## 1. Introduction

Body composition is an important parameter of physical fitness among competitive rowers. Excessive fat mass (FM) lowers speed production by increasing drag when propelling the boat across the water. On the other hand, fat-free mass (FFM) plays a significant role in the expression of strength and power with each stroke [1,2,3]. Indeed, both metrics are important characteristics of rowing performance, and are often more important than body mass alone [4]. It is not surprising that an optimal FFM-to-FM ratio at any given body weight may be the most important consideration when relating body composition to performance among competitive rowers [4]. In this regard, the 2 km (2k) timed trial on an indoor rowing ergometer is commonly used to measure rowing performance [5,6]. Because the needed equipment for this test is on land, ecological validity is of concern. To account for this potential limitation, the timed trial is often scaled to account for body mass, which better reflects on-water performance during real-life competition [7]. The outcome of the test relates to body composition, as athletes that possess lower FM and higher FFM values have been shown to produce more power during the timed trial and complete the 2k distance faster [4,8]. However, the majority of the previous research has focused on male subjects and hence, limited research exists in female athletes. This is an important consideration due to the sex-related differences in body composition and fat distribution patterns. Thus, specific research aimed to examine the relationship between body composition and rowing-related performance in female athletes is needed.

Though there are a mulitude of available body composition assessment methods for field settings, such as the skinfold technique, but many are either not readily available or require specialized training to acquire an accurate reading. Furthermore, technical skill varies between practitioners when using many practical methods and hence, inter-rater reliability of the most common techiques has been a concern [2,9]. Therefore, a simpler assessment method that decreases the potential technician-related error would be of interest to athletes, coaches, and trainers. 

Recently, a method of measuring body composition from a single 2-dimensional (2D) image was proposed for use within field settings [10]. The proprietary automated process predicts body volume by measuring the widths and lengths of various anatomical landmarks within a picture. The system is available commerically as a smartphone application referred to as “made health and fitness”. Previous investigation compared this novel method to underwater weighing in a large sample from the general, healthy population [10]. The findings demonstrated a 0.99 correlation coefficient and no significant mean differences in estimates of body volume [10], in which the smartphone application converts to estimates of FM and FFM. Because of the simplicity of assessment, the new method has potential to be commonly used among practitioners for assessing body composition in athletes. However, whether the body composition metrics derived from the 2D image system relate to rowing performance, is unknown. Therefore, the purpose of this study was to determine the relationship between 2k performance and measures of body composition (i.e., FM and FFM) measured with the novel 2D imaging system, via the commercially available smarphone application, in competitive female rowers. The hypothesis was that 2k rowing performance would be related more strongly to FFM when compared to FM. 

## 2. Materials and Methods

### 2.1. Study Design

FM and FFM was assessed with a newly developed 2D image processing system in a group of collegiate female rowers (*n* = 19). Each participant also performed the 2 km rowing trial (2k). The completion of all testing procedures occurred before the competitive season and required two data collection sessions that were separated by no more than 72 h as follows: (1) body composition assessment and (2) 2k performance. Session 1 took place in the exercise physiology laboratory, while session 2 took place at the rowing training facility. Comparisons were made through correlational procedures with the body composition measures serving as the independent variables and 2k performance as the dependent variable.

### 2.2. Participants

Nineteen female NCAA Division 1 athletes (ages = 20.3 ± 1.0 years, weight = 73.8 ± 8.3 kg, height = 172.7 ± 4.7 cm) from the University’s rowing team participated in this study. Based on previous research in male collegiate rowers that indicated lean mass was strongly correlated with 2k rowing time, an a priori power analysis indicated that a minimum sample of 9 participants would be needed to detect a correlation of 0.8 with 80% power and an alpha level of 0.05 [11]. Thus, the sample size of the current study was deemed as adequate. The University’s Sports Medicine staff medically cleared the participants before performing any of the study procedures. The study was conducted according to the guidelines of the Declaration of Helsinki, and approved by the Institutional Review Board of the University of Alabama (#18-11-1776, approved on 08/01/2019). Informed consent was obtained from all subjects involved in the study.

### 2.3. Procedures

Subjects were instructed to refrain from eating or drinking anything other than water for 3 h prior to data collection, which was confirmed via self-report upon their arrival to the exercise physiology lab. Nude body mass (BM) was measured to the nearest 0.1 kg using a calibrated digital scale (Tanita BWB-800, Tanita©, Arlington Heights, IL, USA). Standing height was measured to the nearest 0.1 cm with a stadiometer (SECA 213, Seca Ltd., Hamburg, Germany). 

**Figure 1:**
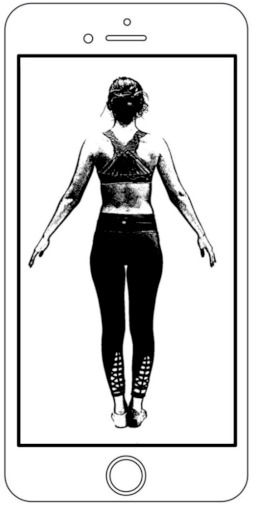
An example of appropriate clothing and body positioning that was used for each scan within the smartphone application’s 2D image analysis system.

For the 2D imaging system, the subjects remained fully clothed while the images were obtained. According to the instructions, the subjects wore “snug-fitting” athletic clothing, such as sports bra and compressive shorts, which allowed the automated processing system to identify the anatomical points of interest (neck, hip, waist, etc.). According to the developers’ preparatory instructions, this is important for an accurate result, as baggy clothing will distort the body’s natural contour which will provide a source of error. However, the research technicians ensured that the clothing each participant wore met the required specifications. If necessary, the subjects were instructed to pull their hair “back” and “up”. The subjects stood in front of a white photography background facing away from the digital camera. Their heels were placed together with the feet flat on the floor and pointed outward at an approximate 60° angle. Participants were required to remain motionless with arms abducted within the coronal plane at a 45° angle away from the torso with palms facing away from the camera. Please see Figure 1 for an example of appropriate clothing and body positioning. A single digital image that included the entire body of the individual was obtained from the rear/posterior view using a 12.9 inch, 64GB iPad Pro, and analyzed using a commercially available smartphone application (www.mymadeapp.com (accessed on 12 October 2020)). To avoid any parallax error, the iPad was positioned on a tripod at approximately 3 m straight from the participant at a level equal to waist-height. The automated system identified a series of anatomical dimensions and an undisclosed proprietary algorithm estimated FM and FFM. One investigator was responsible for taking the 2D image for all participants.

Rowing performance was determined on a rowing ergometer (Concept II, Morrisville, VT, USA). Each subject performed timed trials over 2 kg distances. Data collection for 2k took place inside the rowing team’s training facility, in an area with multiple rowing ergometers that allowed for large scale testing. Prior to the start of the 2k, the athletes warmed up for 30 min as a team. The warm-up consisted of 20 min of rowing at 16, 18, 20, 18, and 16 strokes per min (spm); 4 min at each rate. For the final 10 min, the athletes rowed at a self-selected “goal pace” for 30 s followed by 90 s at 18 spm; this was repeated five times. Upon completion of the 30 min warm up, the ergometer was programmed at a damper setting of “3–4” on the control dial. The athletes were instructed to “simulate an on-water race”, starting with an initial spurt followed by a constant pace at their preferred stroke rate. The total completion time (s) was recorded and performance was expressed as 2k speed (2000 m/total completion time (s)). In addition, 2k speed was adjusted by dividing by body mass (2000 m/total completion time (s)/kilogram) to scale the variable and better reflect performance when rowing in water [7]. As such, the dependent variables of interest in the current study were raw 2k speed and adjusted 2k speed. 

### 2.4. Statistical Analyses

All data were analyzed with Microsoft Excel and SPSS version 26 (Somer, NY, USA). Pearson’s r values were calculated to determine the relationships between the body composition measures and 2k rowing performance. As height was previously associated with rowing performance [12], partial correlations adjusting for height were also performed. To qualify the magnitude of the correlations, an r value between 0 to 0.30 was considered small, 0.31 to 0.49 moderate, 0.50 to 0.69 large, 0.70 to 0.89 very large, and 0.90 to 1.00 near perfect [13]. Data are reported as mean and standard deviations (M ± SD) unless otherwise indicated. Statistical significance for all tests was determined as *p* < 0.05.

## 3. Results

Means ± SD of the studied variables are shown in Table 1.

**Figure 2:**
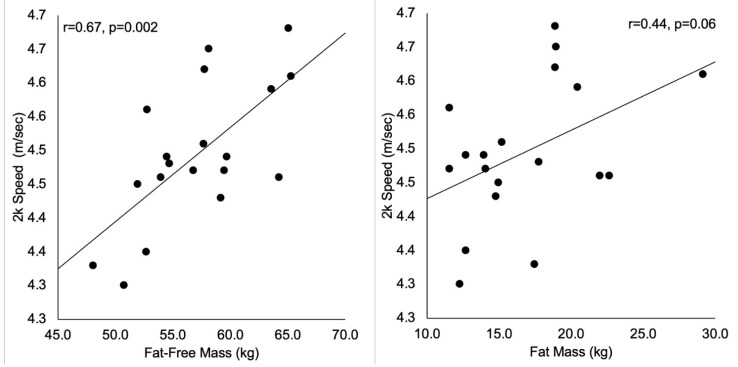
Scatterplots representing the relationships between unadjusted 2 km (2k) speed and the body composition metrics.

A large correlation was observed between FFM and unadjusted 2k speed (Figure 2), such that higher FFM was associated with faster speeds. It should be noted that height was also associated with 2k speed (r = 0.54, *p* = 0.018); however the correlations for FFM remained statistically significant after accounting for the height of the participant (partial r = 0.69, *p* = 0.012). FM was moderately correlated with unadjusted 2k speed; however, this relationship was not statistically significant (*p* = 0.06, Figure 2).

**Figure 3:**
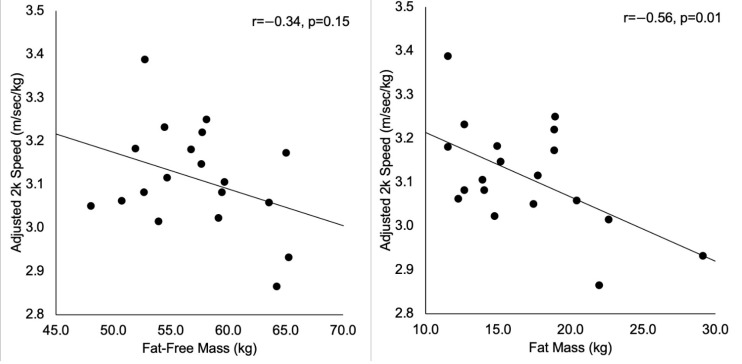
Scatterplots representing the relationships between adjusted 2 km (2k) speed and the body composition metrics.

When adjusted for body weight, 2k performance was not significantly correlated to FFM (Figure 3). However, FM was inversely correlated with adjusted 2k speed (Figure 3), indicating higher FM was associated with lower speeds. This correlation remained statistically significant after accounting for height (partial r = −0.60, *p* < 0.01). 

## 4. Discussion

The study sought to determine the extent to which 2k rowing performance was related to FM and FFM. The hypothesis that FFM would be more strongly related to 2k performance when compared to FM was partially supported. The findings indicated that the 2k timed trial on the rowing ergometer was more strongly correlated with FFM, such that higher FFM was associated with faster 2k speed. In contrast, FM was more strongly related to adjusted 2k performance, which was expressed as meters per min and adjusted for body mass. These findings indicate that higher FM would result in slower speeds, possible due to increased drag. 

Higher height values may translate to an overall larger athlete with longer limbs and trunk lengths, as well as greater horizontal reach. These are important factors toward enhancing mechanical advantage of an athlete producing force against the resistance on the ergometer, thereby increasing propulsion and decreasing 2k time. Thus, taller athletes have been shown to perform better in 2k timed trials [14]. The current study determined the relationships between body composition and rowing performance independent of height with the follow-up partial correlation procedures. The results showed that the significance remained even after accounting for height.

Additionally, larger FFM values are typically found amongst heavier athletes. As such, the body composition metric may be more important than total body weight. Previous research has demonstrated both body weight and FFM significantly contributed to regression equations that predicted maximal oxygen consumption and anaerobic Wingate performance in male rowers [15]. However, the importance of the latter metric was expressed by the authors’ conclusion that improvements in body weight should be achieved by increasing FFM [15]. This is supported by the present findings of large and significant correlations between FFM and the unadjusted 2k timed trial. This finding may be explained by greater FFM values corresponding to increased strength and power production [16,17], which are key determinates of 2k rowing performance [16,18]. Indeed, previous research indicated that higher FFM was associated with faster speeds, whereas higher FM was associated with slower speeds, but accounting for each individual tissue component was more strongly related with 2k performance than total body mass [19]. 

Because of the aforementioned reasons, the 2k timed trial was scaled to account for body weight, providing an adjusted 2k speed in meters per second. These results suggest that although FFM appears to hold a stronger relationship with the unadjusted ergometer performance in this study, the findings should not discount the importance of FM because of the observed relationship with adjusted 2k time. FM is considered nonfunctional regarding athletic performance [20,21]; hence, an excessive amount will heighten overall body mass and add unnecessary weight to the boat. As a consequence, FM potentially adds drag resistance making it more difficult to propel the boat in the water during competition [22]. In addition, improvements in both FM and FFM following training corresponded to decreased 2k time in competitive rowers [23]. Furthermore, it is well understood that FM is associated with obesity-related disorders [24]. Thus, for improved rowing performance and overall health, an optimal FFM-to-FM ratio should be the major focus when designing specific training and dietary practices of athletes. However, practitioners should be aware of issues associated with the female athlete triad when placing too much emphasis on “leanness” [25]. To mitigate associated risks, and in light of current and previous findings regarding the relationship between body composition and rowing-related performance, it may be most important to emphasize increasing FFM among athletes.

FM and FFM were analyzed with a novel 2D image processing system. The research regarding the validity of this new approach has begun to emerge. For instance, Fedewa et al. recently demonstrated that body volume estimates from the same 2D method of the current study displayed an r of 0.99 when compared to the underwater weighing technique in a sample of healthy adults [10]. In addition, when the body volume estimates were combined with total body water measures from bioimpedance spectroscopy for a “field-based” three-compartment model, there were very large correlations (r = 0.96 to 0.99) with the laboratory method for FM and FFM [10]. Moreover, Farina et al. [26] showed no significant differences, strong relationships (R^2^ = 0.91 to 0.95), and a small standard error of estimate values (±2.71 to 2.83 kg) when comparing FM estimates from a different 2D body composition program to dual-energy X-ray absorptiometry. It should be noted that the 2D image system of Farina et al. [26] predicts body volume from the number of pixels comprised by a person’s body within an image. This is a different process than the 2D technique of the current study and Fedewa et al. [10], which estimates body composition by the width of various anatomical dimensions within the image. However, the developing research collectively suggests that 2D imaging techniques may provide accurate and convenient tools for estimating body composition in field settings. As such, the 2D method in the current study should be considered among practitioners for measuring FM and FFM in athletes.

Because of the simplicity of using the 2D image technique, it may be a practical alternative to traditional techniques, especially for providing consistent measures. For example, the skinfold technique is a common field method of body composition. However, issues with inter-rater reliability have been a concern [2]. For instance, Wagner et al. [27] demonstrated that the skinfold method provided very large 95% confidence intervals of intraclass correlations between two technicians, which has been supported by others [28,29]. Because of inconsistencies between technicians, Kispert and Merrifield [29] concluded that the skinfold technique was insufficient for tracking changes in body composition. These previous findings may be due to the fact that the skinfold method requires a degree of technical skill and knowledge to perform correctly, which can vary among practitioners. However, the 2D image technique requires only the ability to take a picture from a smartphone. The built-in processing system automatically populates the measures of FM and FFM. Thus, technician skill is not as much of a requirement as it is with the skinfold method. Though the present study did not assess reliability, it is reasonable to consider that because of the simplicity of taking a 2D image, the system may provide more consistent measures across technicians compared to traditional methods such as measuring skinfolds with calipers. 

There are a number of limitations of the study that should be noted. First, the 2k time trial was analyzed on an indoor rowing ergometer and used to estimate performance in competition by adjusting for body mass. However, such an approach for studying rowing performance is fairly common [18]. Second, the study focused on female rowers. As such, the results should not be generalized to males or other athletic and nonathletic populations. Third, only one field method of body composition was assessed, and as a result, the results of the current study should be replicated in other athletes with additional measures of FFM and FM. Fourth, a multicompartment criterion method was not included. Thus, before widespread use of the 2D image technique, it should be properly validated and compared to a multitude of other field measures within a range of populations. Last, the sample size of only 19 athletes may be considered relatively small for correlational analyses. The athletes in the current study participated in testing as part of their practice schedule but were given the option to allow the researchers to use their de-identified data for research purposes. In order to accommodate the restricted time allotted for weekly practice schedules, additional visits or longer testing sessions to gather missing or incomplete data due to equipment malfunction were not possible. Despite these limitations, ~20 participants presented with complete data, and small sample sizes such as these are common in sports science research with collegiate level athletes. Furthermore, the sample size was considered to be of sufficient power (please refer to the Methods section).

## 5. Conclusions

Recent technological advancements in both digital photography and image analysis have led to the automation of a visual body composition assessment. With the findings of the current study, practitioners should consider choosing the 2D automated image analysis for the assessment of body composition in female athletes. The single 2D image offers a simpler alternative to traditional field methods for estimating FM and FFM in athletes. By utilizing the new technology, the study demonstrated that FFM was significantly correlated to 2k performance in female rowers. However, when controlling for body weight with the adjusted 2k speed, significant correlations between performance and FM were revealed, while FFM became less relevant. Thus, both FM and FFM appear to be independently related to rowing performance. 

## Figures and Tables

**Table 1:** Means ± SD of the studied variables.

Variable	Means ± SD	Minimum–Maximum
Height (cm)	172.5 ± 4.5	161.4–179.1
Weight (kg)	73.9 ± 8.7	63.1–94.5
Body Mass Index (kg/m^2^)	24.8 ± 2.8	21.7–33.1
FM (kg)	16.8 ± 4.5	11.6–29.2
FFM (kg)	56.9 ± 5.0	48.1–65.3
Unadjusted 2k speed (m/s)	4.5 ± 0.1	4.3–4.7
Adjusted 2k speed (m/s/kg)	3.1 ± 0.1	2.9–3.4

FM = fat mass; FFM = fat-free mass; 2k = 2 km rowing time trial.

## Data Availability

The data presented in this study are available on request from the corresponding author.
